# Genetic diversity and population structure of bermudagrass (*Cynodon* spp.) revealed by genotyping-by-sequencing

**DOI:** 10.3389/fpls.2023.1155721

**Published:** 2023-06-08

**Authors:** Lovepreet Singh, Yanqi Wu, James D. McCurdy, Barry R. Stewart, Marilyn L. Warburton, Brian S. Baldwin, Hongxu Dong

**Affiliations:** ^1^ Department of Plant and Soil Sciences, Mississippi State University, Mississippi State, MS, United States; ^2^ Department of Plant and Soil Sciences, Oklahoma State University, Stillwater, OK, United States; ^3^ United States Department of Agriculture – Agricultural Research Service (USDA ARS) Western Regional Plant Introduction Station, Pullman, WA, United States

**Keywords:** single nucleotide polymorphism (SNP) markers, germplasm, genomic diversity, diploid, tetraploid, ADMIXTURE analysis

## Abstract

Bermudagrass (*Cynodon* spp.) breeding and cultivar development is hampered by limited information regarding its genetic and phenotypic diversity. To explore diversity in bermudagrass, a total of 206 *Cynodon* accessions consisting of 193 common bermudagrass (*C. dactylon* var. *dactylon*) and 13 African bermudagrass (*C. transvaalensis*) accessions of worldwide origin were assembled for genetic characterization. Genotyping-by-sequencing (GBS) was employed for genetic marker development. With a minor allele frequency of 0.05 and a minimum call rate of 0.5, a total of 37,496 raw single nucleotide polymorphisms (SNPs) were called *de novo* and were used in the genetic diversity characterization. Population structure analysis using ADMIXTURE revealed four subpopulations in this germplasm panel, which was consistent with principal component analysis (PCA) and phylogenetic analysis results. The first three principal components explained 15.6%, 10.1%, and 3.8% of the variance in the germplasm panel, respectively. The first subpopulation consisted of *C*. *dactylon* accessions from various continents; the second subpopulation was comprised mainly of *C. transvaalensis* accessions; the third subpopulation contained *C. dactylon* accessions primarily of African origin; and the fourth subpopulation represented *C. dactylon* accessions obtained from the Oklahoma State University bermudagrass breeding program. Genetic diversity parameters including Nei’s genetic distance, inbreeding coefficient, and Fst statistic revealed substantial genetic variation in the *Cynodon* accessions, demonstrating the potential of this germplasm panel for further genetic studies and cultivar development in breeding programs.

## Introduction

*Cynodon* L. C. Rich. is a genus consisting of nine warm-season grass species in the Cynodonteae tribe, Chloridoideae subfamily, and grass family (Poaceae) (Harlan et al., 1970; Wu, 2011). *Cynodon* is characterized by a globally extensive distribution and harbors rich genetic diversity. Among these species, common bermudagrass (*C. dactylon* var. *dactylon*) is the most important species economically and ecologically. *Cynodon dactylon* var. *dactylon* is a C_4_ perennial grass used as a turfgrass, forage, and soil stabilizer (Harlan and De Wet, 1969). It has also been reported to be used in ethno-medicinal and traditional medical practices—it is used in Ayurveda, Unani, Nepalese, and Chinese medical systems and has both antiviral and antimicrobial properties (Shendye and Gurav, 2014). *Cynodon dactylon* is a weed in agriculture (Horowitz, 1996; Nelson and Burns, 2006), as well as in various maintained turfgrass settings (McElroy and Breeden, 2006; Uddin et al., 2010). Another important species in the *Cynodon* genus is *Cynodon transvaalensis* Burtt-Davy, commonly known as African bermudagrass. African bermudagrass itself is not widely used for turfgrass due to high water and fertilizer input requirements and poor performance under extreme temperature conditions (Taliaferro, 1992). African bermudagrass does however contain useful traits (i.e., fine texture, high density, and tolerance to low mowing) for turf cultivar development, and it can readily cross with common bermudagrass to generate interspecific hybrids; thus, it is often used in turfgrass breeding programs.

Ploidy levels and chromosome numbers of the genus *Cynodon* vary widely. Flow cytometry studies revealed diploidy to hexaploidy levels in *Cynodon* (Wu et al., 2006; Kang et al., 2008; Gulsen et al., 2009; Jewell et al., 2012; Grossman et al., 2021). Forbes and Burton (1963) confirmed the base chromosome number as 9 in *Cynodon* species. According to the widely accepted revised taxonomy of *Cynodon* (Harlan et al., 1970a), diploidy (2n = 2x = 18) and tetraploidy (2n = 4x = 36) are common but hexaploidy (2n = 6x = 54) is rare. Species including *C. barberi*, *C. dactylon* var. *aridus*, *C. incompletus* var. *incompletus*, *C*. *plectostachyus*, and *C*. *transvaalensis* are predominantly diploid (2n = 2x = 18). Species including *C. arcuatus*, *C. dactylon* var. *dactylon*, *C. dactylon* var. *coursii*, *C. dactylon* var. *elegans*, and *C. dactylon* var. *polevansii* are largely tetraploid (2n = 4x = 36). Other species with both diploid and tetraploid cytotypes are *C. aethiopicus*, *C. dactylon* var. *afghanicus*, *C. incompletus* var. *hirsutus*, *C. nlemfuensis* var. *nlemfuensis*, and *C. nlemfuensis* var. *robustus* (Harlan et al., 1970a). A majority of the common bermudagrass germplasm in breeding programs are tetraploids (Mutlu et al., 2014). Although the classification of *C. dactylon* species as allotetraploid versus autotetraploid is debated (Harlan and De Wet, 1969; Fang et al., 2020; Chaves et al., 2022; Bethel et al., 2006; Harris-Shultz et al., 2010; Guo et al., 2015; Khanal et al., 2017), a recent high-density genetic map developed using genotyping-by-sequencing (GBS) provides a clearer interpretation of the *C. dactylon* allotetraploid structure (Fang et al., 2020).

Polyploidy creates higher genetic diversity in bermudagrass, and it has been reported that genetic diversity within-population was highest at low latitudes (Zhang et al., 2019). Population diversity, structure, and relationships can be studied using DNA markers, which are increasingly used in basic genomic studies and plant breeding programs. In previous studies, genetic diversity in *Cynodon* was largely assessed using traditional molecular markers like amplified length fragment polymorphism (AFLP) (Zhang et al., 1999; Wu et al., 2006), simple sequence repeat (SSR) (Ling et al., 2012; Wang et al., 2013), random amplified polymorphic DNA (RAPD) (Al-Humaid and Motawei, 2004), inter-simple sequence repeat (ISSR) (Farsani et al., 2011; Li et al., 2011), and sequence related amplified polymorphism (SRAP) markers (Wang et al., 2009; Huang et al., 2014; Zheng et al., 2017). Molecular markers have been used to study variation among *Cynodon* species, bermudagrass genotypes, cultivar identification, and off-type confirmation (Caetano-Anollés et al., 1995; Assefa et al., 1999; Zhang et al., 1999; Karaca et al., 2002; Roodt et al., 2002; Wang et al., 2010; Farsani et al., 2011; Dong et al., 2022). The use of genotyping-by-sequencing (GBS) to evaluate off-type grasses in hybrid bermudagrass has also been proposed (Reasor et al., 2018).

Genetic mapping studies are conducted to delineate the framework of chromosomes and provide resources to identify genomic regions that underly traits of interest in bermudagrass. The F_1_ progeny population derived from *C. dactylon* ‘T89’ (4x = 36) × *C. transvaalensis* ‘T574’ (2x = 18) was analyzed to construct genetic maps for each parent (Bethel et al., 2006; Harris-Shultz et al., 2010; Khanal et al., 2017). Using the same population, early results regarding the genetic architecture of canopy height, stolon internode length, length of the longest stolon, and leaf traits (leaf length and leaf blade) were reported (Khanal et al., 2019). Recently, GBS was used in a first-generation, selfed common bermudagrass population, and a high-density genetic map with 3,544 single nucleotide polymorphism (SNP) markers was reported (Fang et al., 2020). More recently, a high-density genetic map for African bermudagrass was created using a GBS approach and QTLs for sod establishment rate were identified (Yu et al., 2021).

Although previous studies provided valuable insights into the genetic diversity, population structure, and genetic architecture of traits in *Cynodon*, most focused on small germplasm collections and accessions that were location or country specific, with low numbers of markers. Individual bi-parental or selfed populations only sample limited allelic diversity of selected parents. Therefore, a *Cynodon* germplasm panel encompassing accessions of worldwide origins evaluated with a large volume of molecular markers would provide novel insights into the genomic diversity of this important genus. As such, a *Cynodon* germplasm panel of 206 accessions consisting of 193 common bermudagrass and 13 African bermudagrass accessions was assembled to study the genetic diversity at the genus level using the GBS approach. The objectives were to obtain high quality SNP markers and to assess the population structure, genetic relatedness, and evolutionary relationship among accessions of the germplasm panel, and to explore their potential for genome-wide association studies.

## Materials and methods

### Plant materials

**Figure 1:**
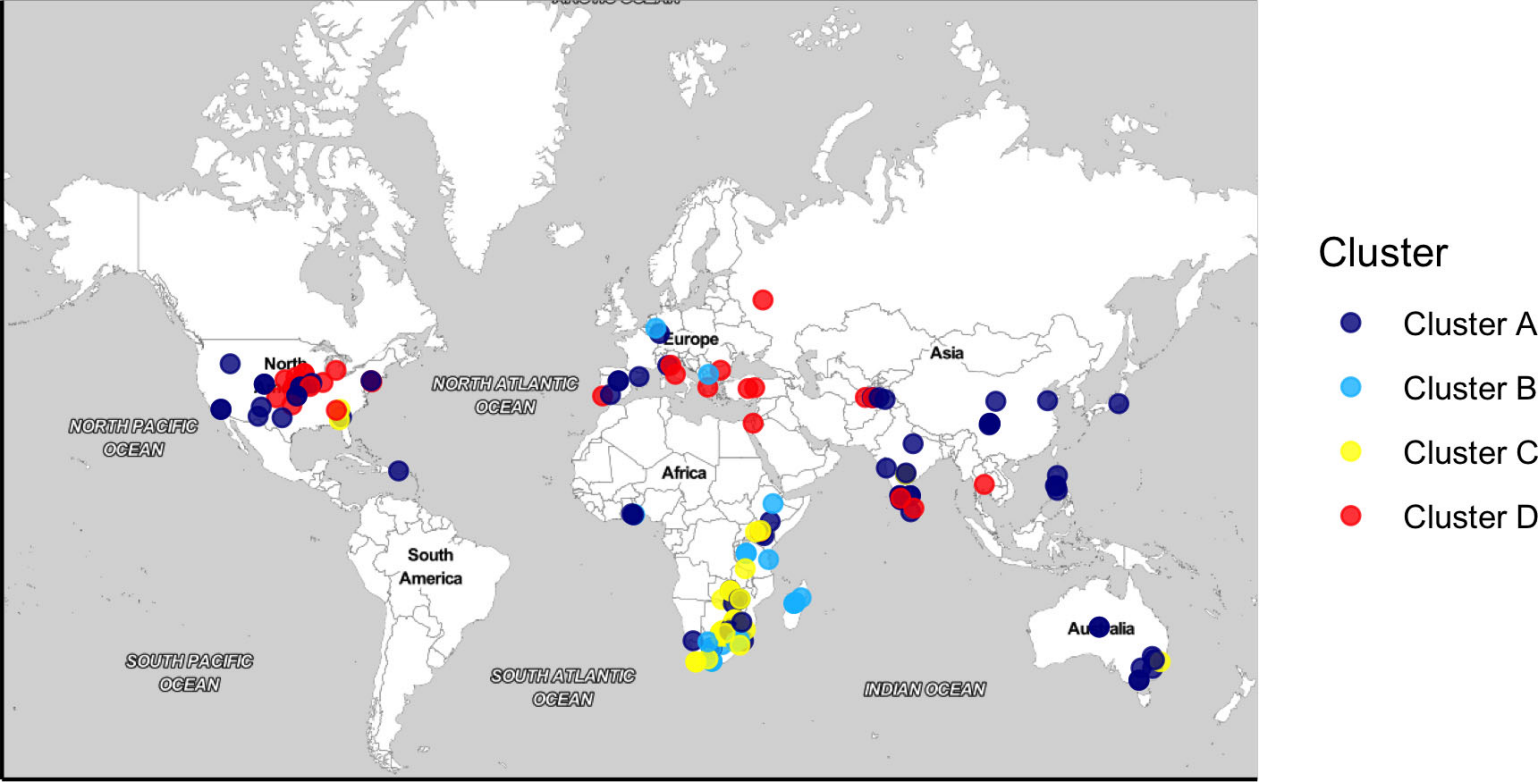
Geographic map of 183 bermudagrass (*Cynodon* spp.) accessions in the germplasm panel. Accessions were colored according to four subpopulations revealed by ADMIXTURE analysis.

A total of 206 bermudagrass accessions (193 common bermudagrass (*C. dactylon*) and 13 African bermudagrass (*C. transvaalensis*) genotypes) were studied (**Table S1**), among which 145 accessions were procured from the USDA National Plant Germplasm System (NPGS), 40 accessions were obtained from the Oklahoma State University (OSU) bermudagrass breeding program, and 21 accessions were from the Mississippi State University (MSU) germplasm collection. The ploidy level of accessions ranged from diploid to tetraploid. These accessions are of worldwide origin (covering five continents) representing different geographical locations (29 countries) and genetic diversity (**Figure 1**).

### DNA extraction, library construction, and genotyping-by-sequencing

The germplasm was cultured in separate containers under greenhouse conditions at Mississippi State University. Plant leaf material was harvested and freeze-dried. Leaf samples were then shipped to the University of Minnesota Genomic Center (UMGC), where DNA extraction, library preparation, and sequencing were performed. Genomic sequencing libraries were prepared following the GBS protocol with *Ape*KI enzyme (Elshire et al., 2011). A total of 206 unique barcodes (*i.e.*, corresponding to 206 bermudagrass accessions) were ligated to fragmented DNA sequences. DNA libraries were sequenced with 101 bp single end reads on an Illumina Novaseq SP platform at UMGC.

### SNP calling

Raw sequence data were investigated for base quality using FastQC v0.11.7 (https://www.bioinformatics.babraham.ac.uk/projects/fastqc/). All GBS libraries were sequenced with high-quality reads. The raw GBS data generated by the UMGC did not have inline barcodes, but instead contain variable length padding sequences (0–10 bp) added in front of the enzyme cut site. Therefore, a Perl script (gbstrim.pl, https://bitbucket.org/jgarbe/gbstrim/src/master/) was used to trim padding sequences. The SNPs were then called *de novo* using the UNEAK pipeline (Lu et al., 2013) of TASSEL 3 standalone. Calling parameters included an error tolerance rate of 0.03, a minor allele frequency of 0.05, and a minimum call rate of 0.5 (i.e., no more than 50% of the bermudagrass accessions in the germplasm panel should have missing data at a given data point).

### Population structure and genetic diversity analysis

Data were analyzed using the software ADMIXTURE (Alexander et al., 2009), TASSEL 5.2.77 (Bradbury et al., 2007), adegenet (Jombart et al., 2010; Jombart and Ahmed, 2011), ape (Paradis et al., 2004), pegas (Paradis, 2010), and customized R scripts for plot visualization. To evaluate the hierarchical population structure, the maximum likelihood-based estimation of admixed ancestry with K (subpopulations) ranging from 1 to 10 was conducted in ADMIXTURE v1.3 (Alexander et al., 2009). The optimum number of subpopulations was determined based on a five-fold cross-validation (CV) at K with a minimum CV error. *Cynodon* accessions were assigned into subpopulations (K1-K4) based on its inferred ancestry from ADMIXTURE. In brief, a *Cynodon* accession was assigned to a given population if its ancestry fraction from this subpopulation was highest among four subpopulations. Pedigree relationship and ancestry coefficients (Q) from the optimum K were used for visualization of population structure. Principal component analysis (PCA) was also conducted in TASSEL 5.2.77 (Bradbury et al., 2007), and the resulting PC matrix was visualized in R with customized scripts. An identity by state (IBS) matrix was calculated in TASSEL, and a neighbor joining (NJ) tree was visualized in Interactive Tree Of Life (iTOL) v5 (Letunic and Bork, 2021). Based on the results of population structure analyses, fixation index (*Fst*) between subpopulations were calculated using the adegenet package (Jombart et al., 2010; Jombart and Ahmed, 2011). Observed heterozygosity (Ho), expected heterozygosity (He), number of private alleles, inbreeding coefficients (Fis), Nei’s genetic distance and Fst statistics were calculated using the hierfstat package (Goudet, 2005; Goudet and Jombart, 2015).

## Results

### SNP calling

A total of 600,380,494 reads were generated, with a mean of 2.9 million reads per sample. After trimming padding sequences, a total of 537,127,057 reads were retained. The UNEAK pipeline identified a total of 536,993,374 reads that contained an *Ape*KI enzyme cut site remnant and a barcode sequence. After variant calling, a total of 37,496 raw SNPs were obtained with minor allele frequency of 0.05 and minimum call rate of 0.5. Across these SNPs, 23,324 (62.20%) have transition substitutions, whereas 14,172 (37.79%) have transversions—a transition to transversions ratio (Ts/Tv) of 1.64:1. The frequency of transitions and transversions observed is given in **Table S2**.

Across 37,496 SNPs, missing data rate ranged from 0 to 50%, with an average missing data rate of 32.68%. The average minor allele frequency across all SNPs was 0.13. Among 206 *Cynodon* accessions, missing data rate ranged from 14% to 40%. Although missing data pose a problem, high number of SNPs is desirable in genetic diversity analyses and could partially offset the negative effect of missing data.

### Genetic diversity and population structure

**Figure 2:**
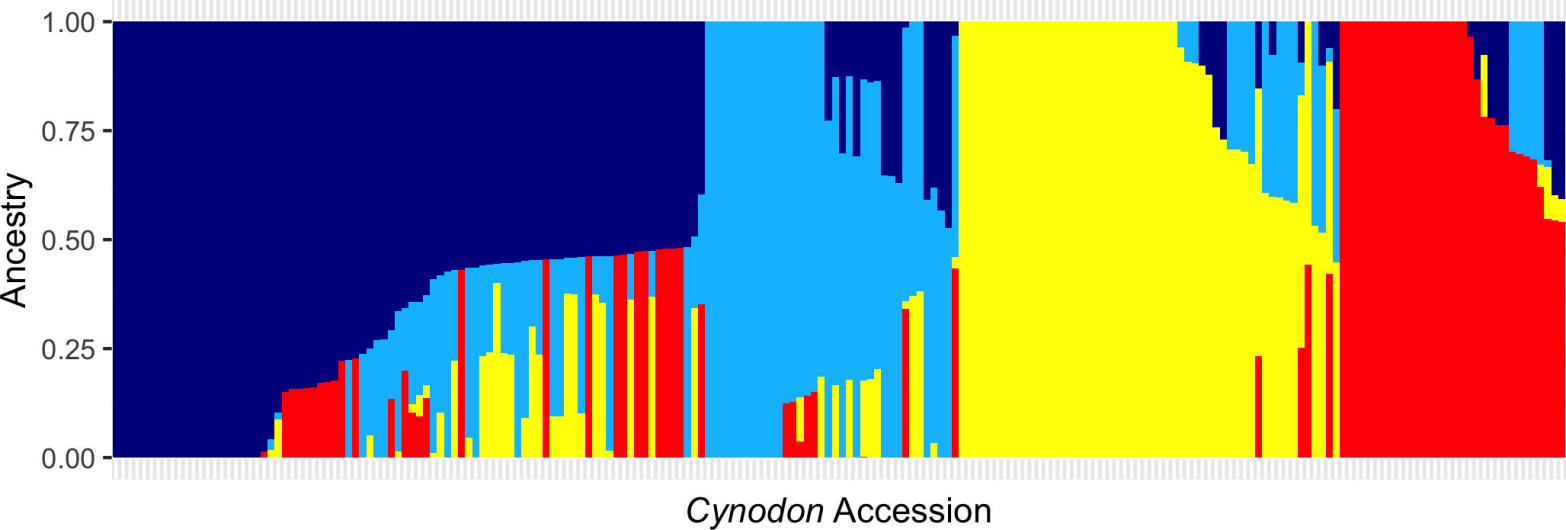
Bar plots from ADMIXTURE analysis. A germplasm panel of 206 *Cynodon* accessions were clustered into four subpopulations (K=4) based on 37,496 genome-wide SNP markers. Dark blue represents ancestral population 1, light blue represents ancestral population 2, yellow represents ancestral population 3, and red represents ancestral population 3.

One of the major objectives of this study was to assess the relationship among 206 accessions and determine the overall population structure. A total of 37,496 SNPs were analyzed in ADMIXTURE and PCA. ADMIXTURE revealed four bermudagrass subpopulations (K=4) based on maximum likelihood clustering model and five-fold cross-validations, which were designated as subpopulations 1–4 (**Figure 2**; **Figure S1**). In brief, subpopulation 1 (84 accessions; 40.7% of the germplasm panel) was comprised of C. *dactylon* accessions collected from seventeen different countries ranging across Africa, Asia, North America, Australia, and Europe (**Table S1**). Two *C. transvaalensis* accessions (PI 647879 and PI 286584) were also assigned to subpopulation 1, suggesting admixture or possibly misidentification of these two accessions. Subpopulation 2 consisted of 36 (17.4%) accessions—22 of African origin, 12 from the United States, and two from Europe. In this cluster, 9 out of 13 C*. transvaalensis* accessions clustered with 27 C*. dactylon* accessions. The third cluster (subpopulation 3) contained 54 (26.2%) accessions—52 of which were *C. dactylon* and two were *C. transvaalensis* (PI 289922 and PI 647878). Again, hybridization or misidentification should be considered for these two *C. transvaalensis.* A majority of the accessions in subpopulation 3 were from five African countries, with two from the U.S., one from India, and one from Australia. The fourth cluster (subpopulation 4) contained the remaining 32 (15.5%) accessions of solely *C. dactylon*. In subpopulation 4, 17 accessions collected in the United States by the Oklahoma State University turfgrass breeding program grouped with 14 accessions collected in neighboring European and Asian countries maintained at the USDA NPGS (Griffin, GA, USA).

**Figure 3:**
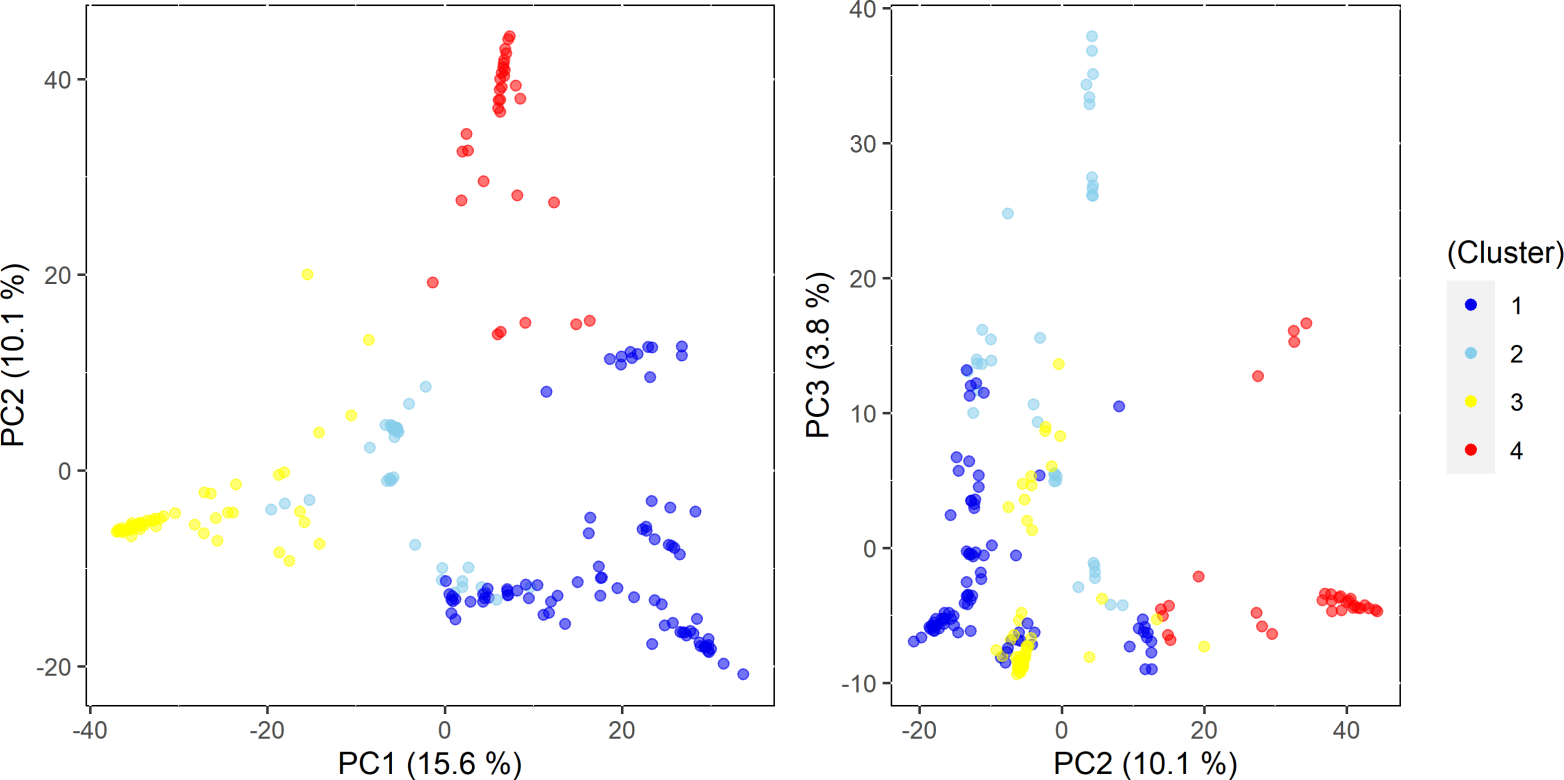
Principal Component (PC) plot extracted from genome-wide SNP markers for this panel and first three principal components explained 15.6%, 10.1%, and 3.8% of the variance in the germplasm panel.

**Figure 4:**
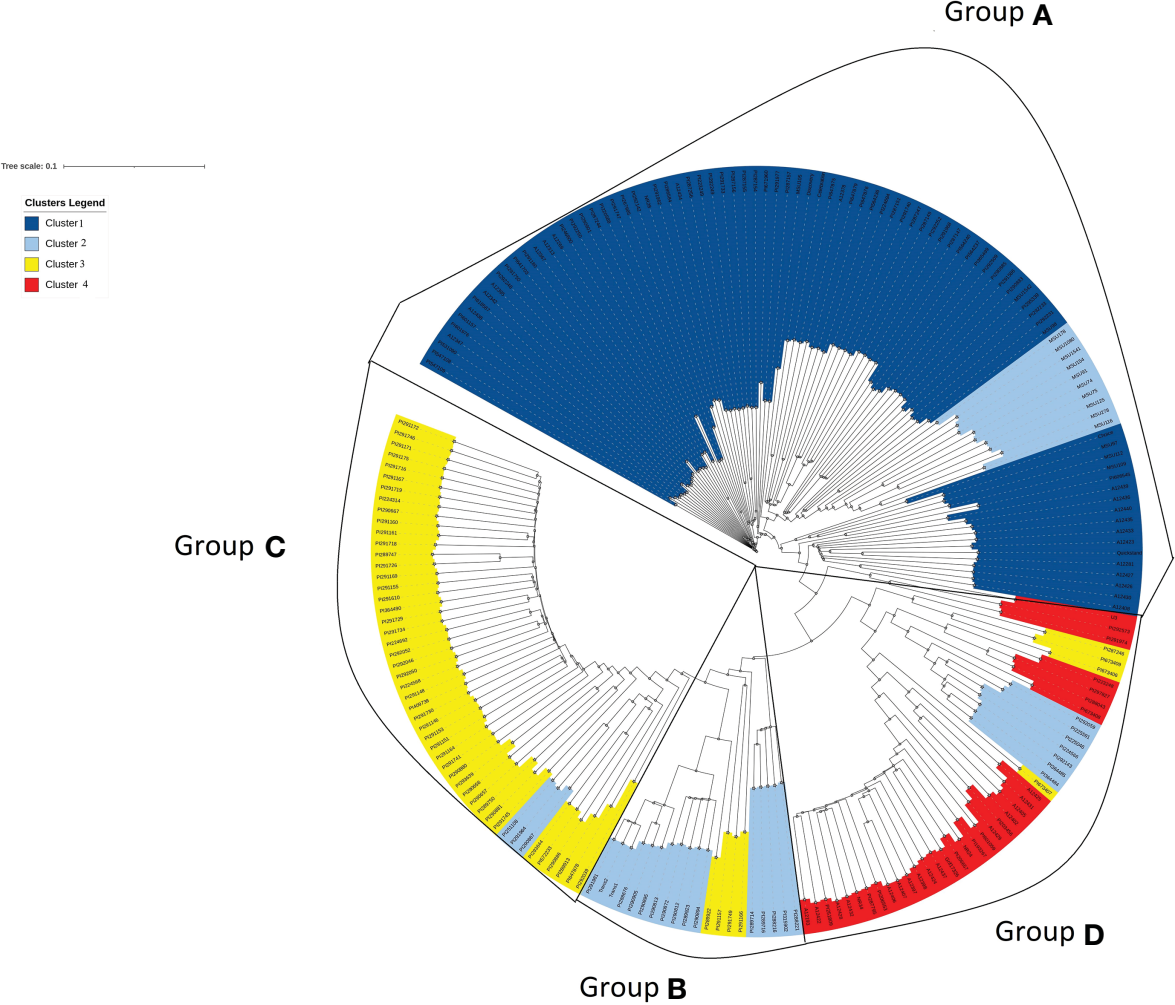
Phylogenetic tree exhibiting relatedness and evolutionary relationship of accessions in the *Cynodon* germplasm panel. Accessions were colored according to four subpopulations (1–4) revealed by ADMIXTURE analysis. Four distinct groups **(A–D)** were marked according to phylogenetic clusters and detailed comparison between ADMIXTURE results and phylogenetic analysis indicated that groupings of accessions were largely consistent. Each branch end represents a *Cynodon* accession.

Similar patterns were found in PCA and phylogenetic analyses (**Figures 3**, **4**), corroborating K=4 in this *Cynodon* germplasm panel. Principal component (PC) analysis indicated that PC1, PC2, and PC3 explained 15.6%, 10.1%, and 3.8% of the genetic variation in the panel, respectively (**Figure 3**). Although there is admixture observed in the PCA analysis (**Figure 3**), the four clusters can still be distinctly observed. The relatedness and evolutionary relationship of *Cynodon* accessions are shown in **Figure 4**. The four groups in the phylogenetic tree were named as A–D (note that some inconsistencies were present but that these generally correspond to 1–4 subpopulations from ADMIXTURE, in corresponding alphanumerical sequence). Detailed comparison between ADMIXTURE results and phylogenetic analysis indicated that groupings of accessions were largely consistent, except for a few differences and for subpopulation 2 (**Figure 4**). In brief, all the accessions of subpopulation 1 from ADMIXTURE also grouped together in the phylogenetic group A, together with the ten *C. dactylon* accessions from the Mississippi State University turfgrass breeding program, which were assigned to subpopulation 2 in ADMIXTURE. The group B in the phylogenetic tree corresponded to ADMIXTURE subpopulation 2 but contained three accessions from subpopulation 3. The group C of phylogenetic tree was found to be consistent with ADMIXTURE subpopulation 3, along with three accessions from subpopulation 2. The phylogenetic group D corresponds to ADMIXTURE subpopulation 4 but also has four accessions from subpopulation 3 and seven accessions from subpopulation 2.

Fixation index (*Fst*) evaluates genetic differences between subgroups within a population. In plants, Fst values greater than 0.15 are considered to significantly differ while values below 0.05 are considered insignificant (Hartl et al., 1997; Frankham et al., 2002). Fixation index (Fst) value between subpopulations 3 and 4 is 0.4895 (**Table 1**). Subpopulation 3 mainly consisted of accessions of African origin (tropical regions), while subpopulation 4 contained accessions primarily from the OSU breeding program; thus, most of the OSU accessions in this study were collected from the US Midwest (temperate regions). These high genetic differences observed between subpopulations 3 and 4 are intriguing, which may suggest that the OSU *Cynodon* accessions could have been enriched for local selective advantages, such as cold tolerance genes, that shaped the genetic difference between these germplasm materials and other *Cynodon* accessions. Pairwise Fst comparison aligned with Nei’s genetic distance (**Table 1**).

**Table 1:** Pairwise fixation index (Fst, lower diagonal) and Nei’s genetic distance (upper diagonal) among four subpopulations in the bermudagrass germplasm panel.

	Subpopulation 1	Subpopulation 2	Subpopulation 3	Subpopulation 4
Subpopulation 1*		0.2016	0.3638	0.3341
Subpopulation 2	0.2248		0.2336	0.3437
Subpopulation 3	0.3771	0.2633		0.4533
Subpopulation 4	0.3693	0.3931	0.4895	

*Subpopulations were determined based on maximum likelihood clustering model in ADMIXTURE software. Three independent runs and five-fold cross-validation were performed.

Plant breeding practices rely on genetic variation in order to develop new cultivars with different genetic combinations including improved traits. Molecular markers provide reliable tools to evaluate genetic variation in germplasm collections. Various parameters of genetic diversity in this germplasm collection are shown in **Table 2**. The highest number of segregating sites were found in subpopulation 1 (n = 35343), followed by subpopulation 3 (n = 28240), 2 (n = 25907) and 4 (n = 21782). Rich genetic diversity exists in this germplasm panel (**Table 2**). Observed heterozygosity (Ho) in four subpopulations (1-4) were 0.12, 0.08, 0.09, and 0.07, respectively. Expected heterozygosity (He) were 0.22, 0.22, 0.17, and 0.19 in subpopulations 1-4, respectively. Subpopulation 1 contained the highest number of private alleles (30,4230), followed by 21,701 in subpopulation 3, 1,185 in subpopulation 2, and 981 in subpopulation 4 (**Table 2**). Inbreeding coefficients ranged from 0.45 to 0.53 across these four subpopulations.

**Table 2:** Population structure in the bermudagrass (*Cynodon* spp.) germplasm panel and genetic diversity index of the four subpopulations.

Subpopulation*	No. of accessions	Polymorphic Sites	Private Alleles	Ho	He	Fis
Subpopulation 1	84	35343	30423	0.12	0.22	0.45
Subpopulation 2	36	25907	1185	0.08	0.22	0.56
Subpopulation 3	54	28240	21701	0.09	0.17	0.47
Subpopulation 4	32	21782	981	0.07	0.19	0.53

*Subpopulations were determined based on maximum likelihood clustering model in ADMIXTURE software. Three independent runs and five-fold cross-validation were performed.

In table the parameters presented are number of accessions, polymorphic sites, private alleles, observed heterozygosity (Ho), expected heterozygosity (He), number of private alleles, inbreeding coefficients (Fis).

## Discussion

Despite recent advancements in high throughput sequencing technologies, genomic resources remain limited in bermudagrass and many other specialty crops. Most previous studies characterizing genetic variation and diversity in *Cynodon* used traditional markers such as AFLP, ISSR, SRAP and SSRs (Wu et al., 2006; Farsani et al., 2011; Ling et al., 2012; Wang et al., 2013; Zheng et al., 2017). Albeit effective and reproducible, data collection from traditional molecular markers is time-consuming and labor-intensive. In this study, we used genotyping-by-sequencing (GBS) technology and obtained a total of 37,496 high-quality SNP markers to evaluate the genomic diversity in 206 bermudagrass accessions from five continents and 29 countries of the world. GBS has identified genome-wide molecular markers at low cost, and this technology is advantageous for breeders of species like bermudagrass in which limited genomic information is available (Kim et al., 2016; Fang et al., 2020). Although missing data were high in part of the SNPs and/or taxa, high number of SNPs is desirable for genetic diversity assessment and could likely benefit data analyses by surveying more genomic regions.

Previous bermudagrass characterization studies were limited due to the small size or limited geographic coverage of populations studied with small numbers of genetic loci sampled, leading to a limited scope in understanding the genetic structure and variations in *Cynodon*. For example, Wu et al. (2004) surveyed the genetic diversity of 28 accessions from 11 countries while Jewell et al. (2012) studied 690 accessions, all collected from Australia. The present study utilized a large worldwide germplasm collection, presumably overcoming limitations of previous studies. Four genetic groups were identified in this *Cynodon* germplasm panel (**Figures 2**, **3**). Similar results were obtained from ADMIXTURE analysis, PCA, and phylogenetic analysis, which corroborates the validity of the genetic structure in this study. Subpopulation 1 consisted of accessions from different geographic locations (**Figure 2**). Such grouping of genetic materials from different locations was likely due to the exchange of breeding materials between programs and common use of materials from the USDA NPGS, which were originally contributed by multiple *Cynodon* research groups. Therefore, many of the studied *Cynodon* accessions may share common ancestry at various levels.

In this germplasm panel, we included 13 African bermudagrass (*C. transvaalensis*) accessions, many of which clustered in subpopulation 2 with *C. dactylon* of African origin (**Figure 2**). As *C. dactylon* plants are predominantly tetraploids and *C. transvaalensis* are diploids, mixture of *C. dactylon* and *C. transvaalensis* in this subpopulation may shed light on the polyploidization origin of *C. dactylon*. This finding is novel. We were surprised to find the common bermudagrass and African bermudagrass accessions were grouped together in the same cluster instead of forming two separate groupings relative to other common bermudagrass accessions. It is well known that common bermudagrass can readily cross with African bermudagrass. Their hybrids, however, are sterile triploids, which cannot backcross with common bermudagrass nor African bermudagrass, indicating a strong gene flow barrier between the two species, or that the two species have independent evolutionary pathways. One reasonable speculation is that ancestor(s) of modern diploid African bermudagrass may have substantially contributed to the formation of tetraploid common bermudagrass. When whole genome sequences of the two species are available, comparative analysis may validate this hypothesis. In *Miscanthus* (Panicoideae subfamily), two major species, *M. sinensis* (largely diploid) and *M. sacchariflorus* (largely tetraploid) are used for biomass breeding. Genetic analyses in these two *Miscanthus* species revealed unidirectional gradient introgression from diploid *M. sinensis* to tetraploid *M. sacchariflorus* (Clark et al., 2019). This finding also supports the current mainstream hypothesis that Africa is the center of origin of *Cynodon* (Burton, 1948; Cui et al., 2021). In subpopulation 3, three *C. transvaalensis* accessions (PI 251108, PI 291964, and PI 290887) grouped with 46 C*. dactylon* (**Figure 2**). In addition to the possibility of close relatedness between these three *C. transvaalensis* accessions and the *C. dactylon* in this group, other reasons may include potential mislabeling of species and contamination of materials. Inevitably, further detailed investigation of accessions is needed.

In subpopulation 4 (**Figure 2**), most accessions from the Midwest and northeastern regions in the US grouped with accessions from European countries and cold/temperate areas in Asia. Bermudagrass is not native to the US (Taliaferro, 1992). This result suggests that the cold hardy, naturalized germplasm collected in OK, KS, NE, IL, MI, MO, and NJ are genetically similar to cold hardy germplasm in Europe and Asia. The genotype A12193 used in this study was collected on the campus of Michigan State University, East Lansing, MI. Bermudagrass on the campus of Michigan State University was a single clone, which was introduced by W. J. Beal in the 1880s. This bermudagrass is amongst the earliest bermudagrass introduced to the region (Gilstrap, 2002; Gilstrap, 2012). The current study indicates that this bermudagrass accession most likely came from Europe or Asia, instead of Africa as originally speculated (Gilstrap, 2002).

Principal component analysis (PCA), ADMIXTURE and phylogenetic analysis all support that most of African accessions formed a unique cluster with the remaining accessions exhibiting admixture (**Figures 2**–**4**). The distinct grouping of African accessions indicates that unique genetic variations exist in this germplasm and that they may have important implications for *Cynodon* breeding. Due to the non-admixture of African accessions with others, they may not have been widely used in breeding programs to date. Genetic diversity within breeding programs decreases due to selection, small population size, genetic drift, and other factors (Reif et al., 2005; Fu, 2015). These accessions can be used in breeding programs to increase genetic variation. Furthermore, most of the African accessions within this PCA cluster are from four nearby countries (South Africa, Zambia, Zimbabwe, Mozambique) in southern Africa, and one accession is from Kenya (East Africa) (**Table S1**). It is reasonable to hypothesize that other African countries could potentially contribute unique and diverse germplasm as well.

Although *Cynodon* species are predominantly outcrossing, breeding programs have mainly relied on small subset. Observed heterozygosity ranged from 0.07 to 0.12, which were considerably smaller than expected heterozygosity which ranged from 0.17 to 0.22 (**Table 2**). This was corroborated by relatively high inbreeding coefficients (0.45-0.56, **Table 2**). These results further supported the grouping of four subpopulations and were likely due to extensive hybridizations of certain accessions as parental lines.

Results shed new light on the introduction history and origin of unknown accessions of bermudagrass. For example, in subpopulation 2, most of the African accessions clustered together except for 12 accessions from the US and two from Europe. This supports the hypothesis that bermudagrass was introduced to colonial America from Africa (Nelson and Burns, 2006). There is a possibility that many *Cynodon* accessions in North America or other places are still genetically similar to the ones from their African origin. The geographic origin of some accessions was unknown, especially the MSU accessions. Inferences from the ADMIXTURE analysis and phylogenetic tree could shed light on the origin of these accessions (**Figures 2**, **4**). Twelve of the MSU accessions were clustered in subpopulation 2 with African bermudagrass, indicating the origin might be in Africa. Most of the MSU accessions resulted from hybridizations made during the past 30 years, in the breeding program and are full- or half-siblings, and thus they clustered closely together. The subpopulation 2 from ADMIXTURE is spread all over the phylogenetic tree in different groups suggests that this subpopulation is related to other subpopulations or may contain admixture from other subpopulations.

The 37,496 high-quality SNPs used in the current study allow us to survey more genomic regions of the *Cynodon* genome and enhances our understanding of this underexplored genus. Moreover, SNP data across the genome in this study assist in the clarification of *Cynodon* accessions with conflicting information and can help in clarifying the misidentification of accessions. As an example, PI 290812, PI 290813, and PI 290872 accessions are currently named as *C. nlemfuensis* by Grossman et al. (2021) while the USDA database lists them as *C. transvaalensis* Burtt Davy. In this study, these three accessions were grouped closely with *C. transvaalensis* (**Figure 4**), supporting the USDA data curation.

## Conclusions

This study revealed extensive genetic diversity in the two *Cynodon* species important to breeding turfgrass cultivars by exploiting a bermudagrass germplasm panel of worldwide origin. To the best of our knowledge, this study reports the highest number of genome-wide SNP markers in a bermudagrass germplasm study. Strong and distinct genetic structure, as revealed by multiple analyses, indicates rich genetic variation for further improvement and development of new bermudagrass cultivars to reach a new adaptive peak. The grouping of African bermudagrass with common bermudagrass populations originating in Africa is a new finding and suggests significance in evolution and adaptation in the Africa continent. This study reveals that naturalized bermudagrass in the Midwest and Northeast of the US were genetically similar and likely introductions from Europe or Asia. The separate groupings of common bermudagrass accessions provides information to breeders and geneticists aiding in parental line selection for developing breeding populations. This study provides a valuable guide for allele mining of desirable genes for the traits of interest (e.g., abiotic and biotic stress resistance), which will improve bermudagrass breeding and serve as foundation for genetic mapping of important traits.

## Data availability statement

The SNP datasets generated and analyzed in this study are available in the Figshare repository: https://figshare.com/articles/dataset/Genomic_Diversity_Bermudagrass/21941333. R scripts used in data analysis and visualization are available on Github: https://github.com/SinghLovepreet2363/Bermudagrass_GenomicDiversity.

## Author contributions

LS and HD conceptualized the study, analyzed the data, drafted the manuscript. YW contributed to the germplasm collection, assisted in interpreting the results and edited the manuscript. MW, JM, BS, BB edited and reviewed the manuscript. HD obtained the funding for the project. All authors contributed to the article and approved the submitted version.
