# Supplementary material for: Genetic diversity and population structure of bermudagrass (Cynodon spp.) revealed by genotyping-by-sequencing
Source: Front Plant Sci. 2023 Jun 8;14:1155721. doi: 10.3389/fpls.2023.1155721 (PMC10285298; doi:10.3389/fpls.2023.1155721)

Supplementary Material

**
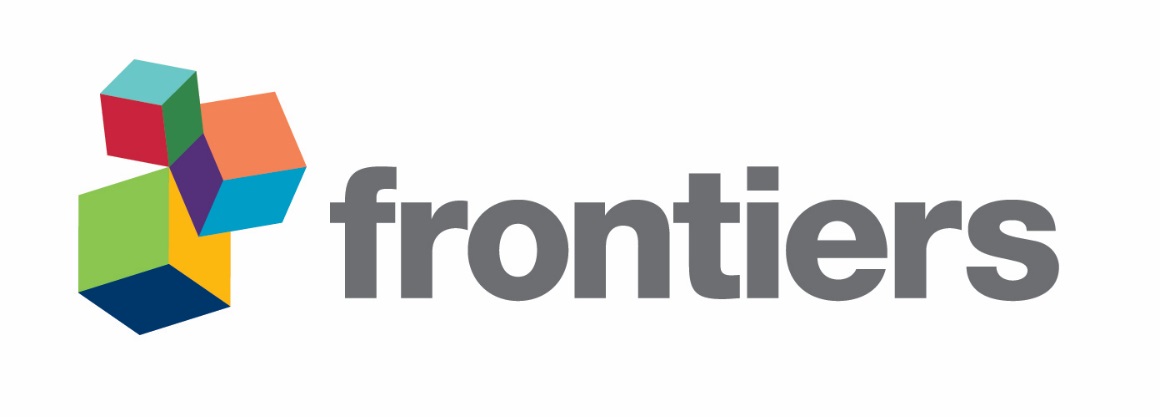
**

**Supplementary Tables**

Supplementary Table S1. Dataset contains source information (ID, species, ploidy, program, origin, genome size) of all 206 *Cynodon* accessions used in the study. Q values from ADMIXTURE analysis are available in Sheet 2 of Table S1.

Supplementary Table S2. Frequency of Transitions and Transversions observed in SNP dataset

| Transitions |  | Transversions |  |
| --- | --- | --- | --- |
| A/G | 11458 | A/C | 3431 |
| G/A | 173 | G/T | 3370 |
| C/T | 11501 | A/T | 2809 |
| T/C | 192 | G/C | 76 |
| Total | 23324 | C/G | 4442 |
|  |  | T/A | 44 |
|  |  | Total | 14172 |

**Supplementary Figure**

Supplementary Figure S1. Cross-validation figure from Admixture


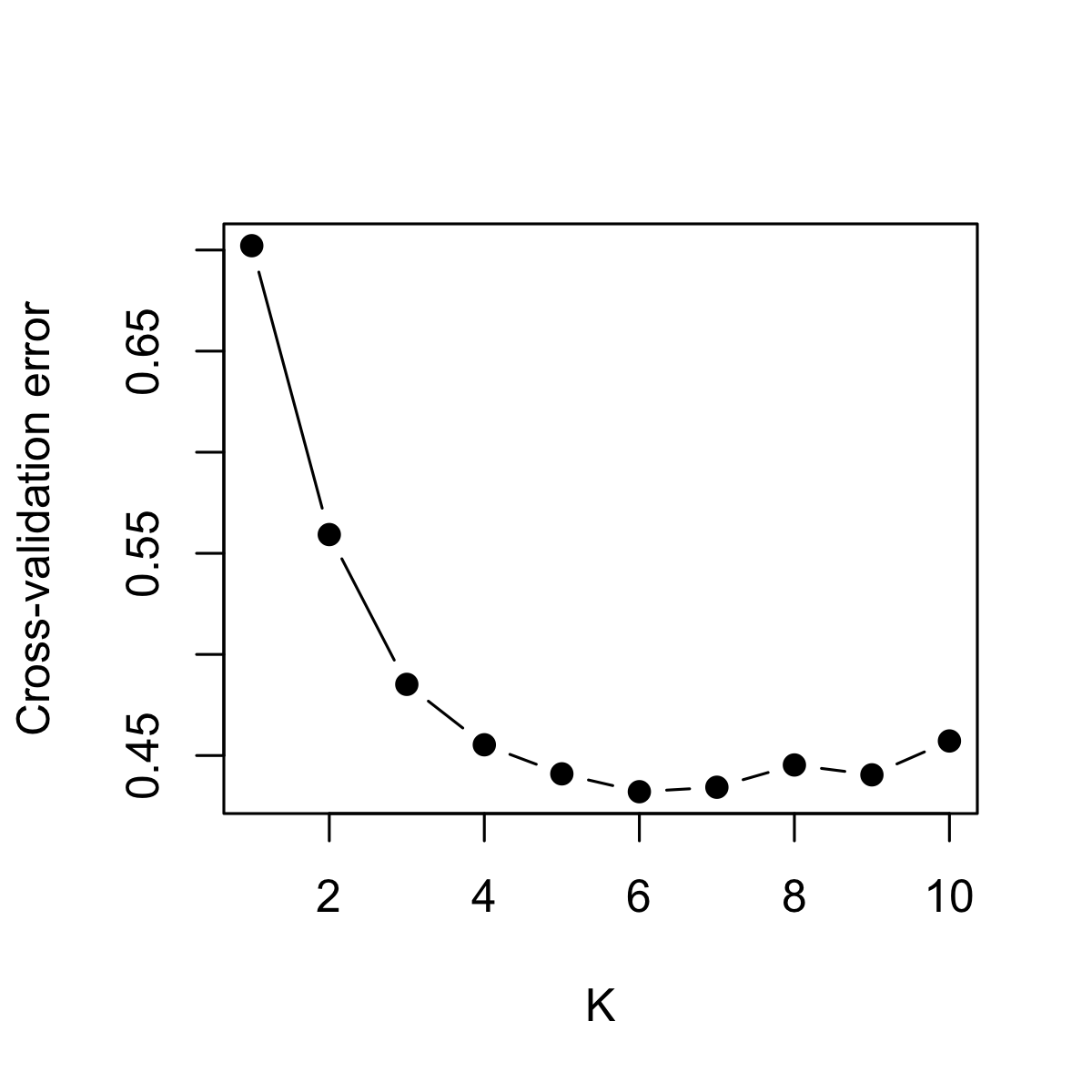

Supplement: Supplementary file 1 [file DataSheet_1.docx]
